# Multiple Inhibitory Mechanisms of DS16570511 Targeting Mitochondrial Calcium Uptake: Insights from Biochemical Analysis of Rat Liver Mitochondria

**DOI:** 10.3390/ijms26062670

**Published:** 2025-03-16

**Authors:** Akiko Yamada, Akira Watanabe, Atsushi Nara, Tsubasa Inokuma, Masatake Asano, Yasuo Shinohara, Takenori Yamamoto

**Affiliations:** 1 Department of Pathology, Nihon University School of Dentistry, Chiyoda-ku, Tokyo 101-8310, Japan; 2 Institute for Genome Research, Tokushima University, Tokushima 770-8503, Tokushima, Japan; 3 Graduate School of Pharmaceutical Sciences, Tokushima University, Tokushima 770-8503, Tokushima, Japan; 4 Division of Molecular Target and Gene Therapy Products, National Institute of Health Sciences, Kawasaki 210-9501, Kanagawa, Japan

**Keywords:** DS16570511, inhibitor, mitochondrial calcium uniporter, rat liver

## Abstract

Mitochondrial calcium (Ca^2+^) uptake plays a key role in mitochondrial physiology and disease development. This process is regulated by the mitochondrial calcium uniporter (MCU) complex. DS16570511 is a membrane-permeable drug that inhibits mitochondrial Ca^2+^ uptake, although its inhibitory mechanisms remain unclear. In this study, we evaluated the effects of DS16570511 on various mitochondrial functions through biochemical analyses. We found that DS16570511 affects multiple mitochondrial functions and exhibits variable potency in inhibiting individual processes. Specifically, DS16570511 not only inhibits MCU, its initially reported target, but also respiratory chain complexes and F_o_F_1_-adenosine triphosphatase/adenine nucleotide translocator, particularly respiratory chain complex II. Furthermore, the carboxyl group at the molecular terminus of DS16570511 plays a critical role in its inhibitory effects on mitochondrial Ca^2+^ uptake through respiratory chain complex II inhibition. These findings enhance our understanding of the mechanisms by which DS16570511 inhibits mitochondrial Ca^2+^ uptake and provide valuable insights for the clinical application of mitochondrial Ca^2+^ uptake inhibitors.

## 1. Introduction

In eukaryotes, mitochondrial calcium (Ca^2+^) uptake plays a critical role in regulating processes such as respiration and cell death [1,2,3]. Excessive Ca^2+^ accumulation in the mitochondria triggers the opening of the mitochondrial permeability transition pore, increasing inner membrane permeability. This disruption leads to cell death through apoptosis or necrosis and contributes to various conditions and diseases, including ischemia–reperfusion injury, muscular dystrophies, neurodegenerative disorders, and cancer [4,5,6,7,8]. Ca^2+^ ions are selectively transported from the cytosol into the mitochondria via the mitochondrial calcium uniporter (MCU) complex in a membrane potential-dependent manner [9,10,11]. The MCU complex consists of several components, including MCU [12,13], mitochondrial calcium uptake proteins 1–3 (MICU1–3) [14,15], MCU regulator 1 (MCUR1) [16], an MCU isoform (MCUb) [17], and the essential MCU regulator (EMRE) [18].

Studies on selective MCU inhibitors are crucial for understanding its function, and numerous inhibitors have been investigated. Ruthenium red was the earliest known inhibitor of Ca^2+^ uptake [19], and its more potent analog, ruthenium 360, was named for its maximum absorption at 360 nm [20]. Ruthenium 360 strongly inhibits Ca^2+^ uptake, with a 50% inhibitory concentration (IC_50_) of 184 pM [21]; however, its high hydrophilicity prevents it from penetrating cell membranes, limiting its use in functional analyses of MCUs in cells and its potential for clinical application. To address this limitation, a cell membrane-permeable derivative, ruthenium 265, was designed [22]. Despite its improved properties, intraperitoneal administration of ruthenium 265 in mice caused seizure-like movements, raising concerns about its clinical safety [23,24]. Subsequently, Arduino et al. identified mitoxantrone, an anthraquinone anticancer drug, as an MCU inhibitor [25]; however, its use is limited due to its cardiotoxic side effects.

**Figure 1:**
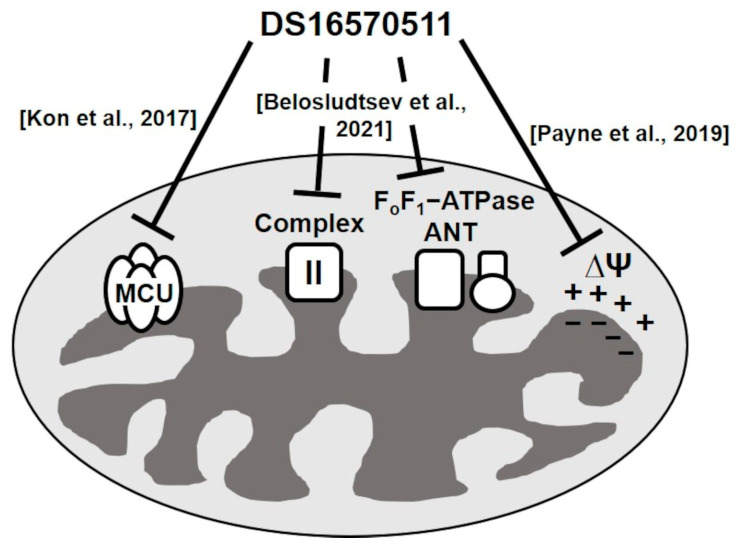
Summary of DS16570511’s effects on mitochondrial functions in previous studies. Kon et al. reported that DS16570511 is an MCU-specific inhibitor [26], whereas Payne et al. revealed that DS16570511 affects the formation of mitochondrial membrane potential [27]. Belosludtsev et al. reported that DS16570511 inhibits the activity of respiratory chain complex II, F_o_F_1_-ATPase, and ANT [28]. However, the potency of these inhibitory effects has not been evaluated previously; moreover, the relationship between these inhibitory effects and mitochondrial calcium uptake remains unknown. Therefore, the mechanism by which DS16570511 inhibits mitochondrial calcium uptake remains unclear.

Kon et al. reported a membrane-permeable MCU inhibitor, DS16570511 [26]. DS16570511 was shown to inhibit Ca^2+^ uptake in HEK293A cells and rat heart mitochondria without affecting the mitochondrial membrane potential, which is the driving force for Ca^2+^ uptake. Moreover, it inhibited Ca^2+^ uptake promoted by the overexpression of MCU or MICU1, suggesting that MCU or MICU1 serves as the molecular target of DS16570511. The specificity of DS16570511′s inhibitory effect has not been thoroughly investigated, but it has been classified as an MCU-selective inhibitor. However, Payne et al. later reported that DS16570511 affects mitochondrial membrane potential [27]. Belosludtsev et al. further confirmed that DS16570511 influences membrane potential in brain mitochondria and impacts the function of respiratory chain complex II and F_o_F_1_-adenosine triphosphatase (ATPase)/adenine nucleotide translocase (ANT) [28]. These findings suggest that DS16570511 acts on multiple proteins within the inner mitochondrial membrane (Figure 1). Despite these insights, the specificity of DS16570511′s inhibitory activity remains unclear. In this study, we analyzed the effects of DS16570511 on mitochondrial functions under various conditions and compared the extent of mitochondrial Ca^2+^ uptake inhibition associated with these effects to elucidate the mechanism by which DS16570511 inhibits mitochondrial Ca^2+^ uptake. Furthermore, we identified the functional group of DS16570511 involved in this mechanism. The findings of this study provide valuable insights for the clinical development of DS16570511 and its analogs for treating diseases involving mitochondrial Ca^2+^ uptake.

## 2. Results

### 2.1. Inhibition of Mitochondrial Ca^2+^ Uptake by DS16570511

**Figure 2:**
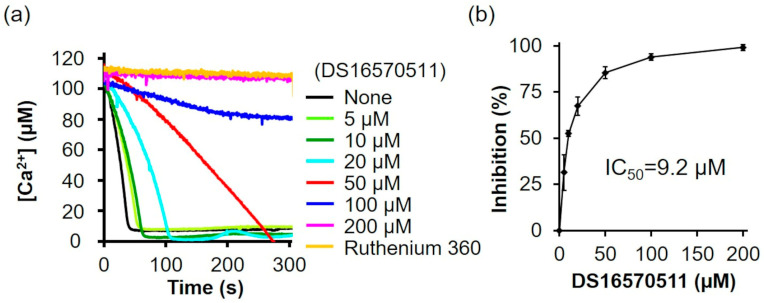
Effect of DS16570511 on mitochondrial Ca^2+^ uptake activity in rat liver mitochondria. (**a**) Mitochondrial Ca^2+^ uptake activity was measured in isolated rat liver mitochondria. Mitochondria were added to Pi medium containing the indicated concentrations of DS16570511 or ruthenium 360, and changes in extramitochondrial Ca^2+^ concentration over time were analyzed based on Fluo-5 fluorescence intensity. Representative traces are shown. (**b**) The degree of inhibition was calculated from the Ca^2+^ uptake rate during the first 30 s after adding mitochondria. Data are expressed as 0% inhibition in the absence of DS16570511 (none) and 100% inhibition in the presence of ruthenium 360. Results are presented as the mean ± standard deviation of independent experiments (*n* = 3).

DS16570511 was synthesized as described by Kon et al. [26] (Supplementary Information). To confirm that the synthesized DS16570511 exhibited the same efficacy as previously reported, we evaluated its inhibitory effect on mitochondrial Ca^2+^ uptake using mitochondria isolated from rat liver and found that the Ca^2+^ uptake rate decreased in a concentration-dependent manner (Figure 2a). The IC_50_ of the synthesized DS16570511, calculated from the relative Ca^2+^ uptake rate (%) during the first 30 s after adding mitochondria, was 9.2 µM (Figure 2b).

### 2.2. Effect of DS16570511 on Mitochondrial Membrane Potential Formation

**Figure 3:**
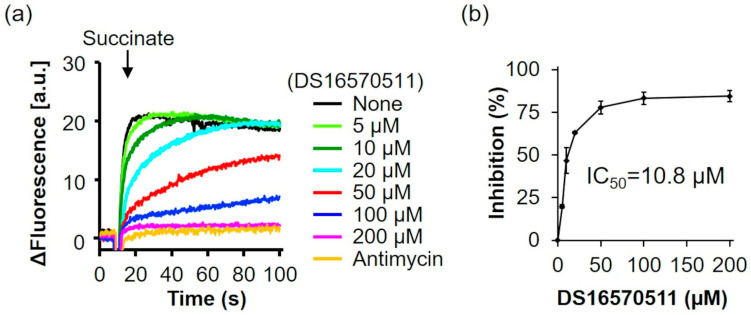
Effect of DS16570511 on membrane potential in rat liver mitochondria. (**a**) Rat liver mitochondria were incubated in Pi medium containing the indicated concentrations of DS16570511 or antimycin. At the time points indicated by arrows, 10 mM succinate was added as a respiratory substrate, and membrane potential was measured using 3 µM DiSC_3_(5) as a probe. Representative traces are shown. (**b**) The degree of inhibition was calculated from the rate of membrane potential formation during the first 5 s after succinate addition, with 0% inhibition in the absence of DS16570511 (none) and 100% inhibition in the presence of SF6847. SF6847, an uncoupler, was used as a control to confirm mitochondrial membrane potential disruption. Data are presented as the mean ± standard deviation of independent experiments (*n* = 3).

Ca^2+^ uptake into the mitochondria is driven by the membrane potential across the mitochondrial inner membrane [9,10,11]. DS16570511 can inhibit Ca^2+^ uptake by disrupting membrane potential formation in addition to directly targeting the MCU complex. Payne et al. and Belosludtsev et al. reported that DS16570511 affects mitochondrial membrane potential, causing either depolarization or hyperpolarization [27,28]. In this study, we measured the effect of DS16570511 on membrane potential formation in rat liver mitochondria and calculated its IC_50_ (Figure 3). DS16570511 inhibited membrane potential formation in a concentration-dependent manner, with an IC_50_ of 10.8 µM, similar to its IC_50_ for inhibiting Ca^2+^ uptake. These results suggest that DS16570511 inhibits Ca^2+^ uptake by suppressing mitochondrial membrane potential formation.

### 2.3. Effects of DS16570511 on the Activity of the Respiratory Chain Complex

**Figure 4:**
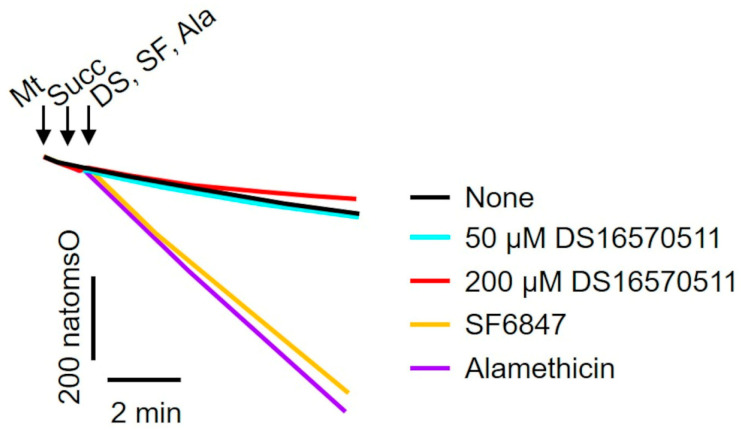
Comparison of the effects of DS16570511 and uncouplers on mitochondrial oxygen consumption. Mitochondria (Mt), 10 mM succinate (Succ), and each compound (50 or 200 µM DS16570511 [DS], SF6847 [SF], or alamethicin [Ala]) were added to Pi medium at the time points indicated by arrows. Changes in the concentration of dissolved oxygen in the reaction medium were measured using an oxygen electrode. Representative traces are shown. “natomsO” denotes the nanomoles of oxygen atoms in the reaction medium.

To investigate the mechanism underlying the inhibition of membrane potential formation, the effect of DS16570511 on mitochondrial oxygen consumption was examined. A decrease in membrane potential could result from either the uncoupling of oxidative phosphorylation or the inhibition of proton gradient formation due to respiratory chain inhibition. To determine whether DS16570511 acts as an uncoupler, its effects on oxygen consumption were compared with those of SF6847, a protonophore, and alamethicin, a channel-forming peptide (Figure 4). Unlike SF6847 and alamethicin, which markedly increased oxygen consumption due to uncoupling, DS16570511 reduced the rate of oxygen consumption. These results indicate that DS16570511-induced membrane potential reduction is not caused by oxidative phosphorylation uncoupling.

**Figure 5:**
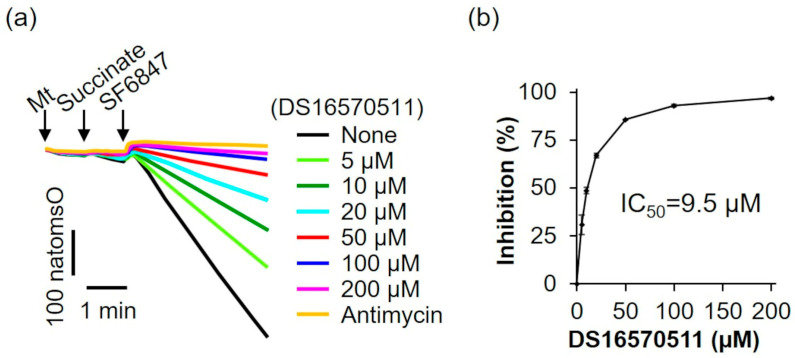
Effect of DS16570511 on oxygen consumption in the presence of succinate. (**a**) Mitochondria (Mt), 10 mM succinate, and 0.1 µM SF6847 were added to Pi medium containing the indicated concentrations of DS16570511 or antimycin at the time points marked by arrows. Changes in dissolved oxygen concentration in the reaction medium were measured, and representative traces are shown. “natomsO” denotes the nanomoles of oxygen atoms in the reaction medium. (**b**) The degree of inhibition was calculated from the rate of oxygen consumption over a 3 min period, measured 30 s after SF6847 addition. Results are expressed as 0% inhibition in the absence of DS16570511 (none) and 100% inhibition with antimycin. Data are presented as the mean ± standard deviation of independent experiments (*n* = 3).

The oxygen consumption rate of rat liver mitochondria was analyzed in the presence of the respiratory substrate succinate (Figure 5a). The addition of SF6847 caused a marked increase in oxygen consumption; however, in the presence of antimycin, this increase did not occur. Similarly, 200 µM DS16570511 inhibited oxygen consumption to the same extent as antimycin, suggesting that DS16570511-induced loss of membrane potential results from its inhibitory effects on the respiratory chain. The IC_50_ for DS16570511′s inhibition of maximal oxygen consumption induced by SF6847 was 9.5 µM (Figure 5b). This value was nearly identical to the IC_50_ for the inhibition of both Ca^2+^ uptake and membrane potential formation (Figure 2b and Figure 3b), strongly indicating that DS16570511 inhibits mitochondrial Ca^2+^ uptake by disrupting membrane potential formation through respiratory chain inhibition.

### 2.4. Effects of DS16570511 on Other Mitochondrial Functions

**Figure 6:**
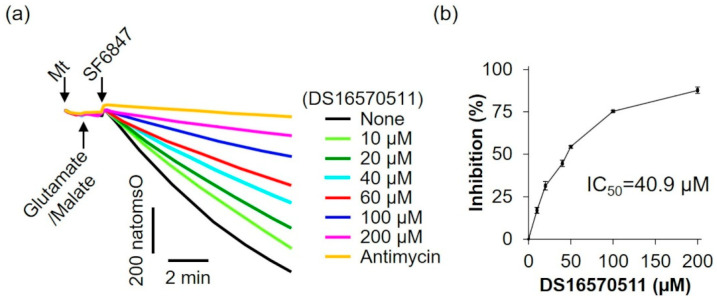
Effect of DS16570511 on oxygen consumption in the presence of glutamate and malate. (**a**) Mitochondria (Mt), 5 mM glutamate, 5 mM malate, and 0.1 µM SF6847 were added to Pi medium containing the indicated concentrations of DS16570511 or antimycin at the time points marked by arrows. Changes in dissolved oxygen concentration in the reaction medium were measured, and representative traces are shown. “natomsO” denotes the nanomoles of oxygen atoms in the reaction medium. (**b**) The degree of inhibition was calculated from the rate of oxygen consumption over a 3 min period, measured 30 s after SF6847 addition. Results are expressed as 0% inhibition in the absence of DS16570511 (none) and 100% inhibition with antimycin. Data are presented as the mean ± standard deviation of independent experiments (*n* = 3).

To further evaluate the effects of DS16570511 on mitochondrial function, its influence on oxygen consumption was examined using glutamate and malate as respiratory substrates, which supply electrons via respiratory chain complex I (Figure 6). DS16570511 inhibited oxygen consumption in a concentration-dependent manner, with an IC_50_ of 40.9 µM.

**Figure 7:**
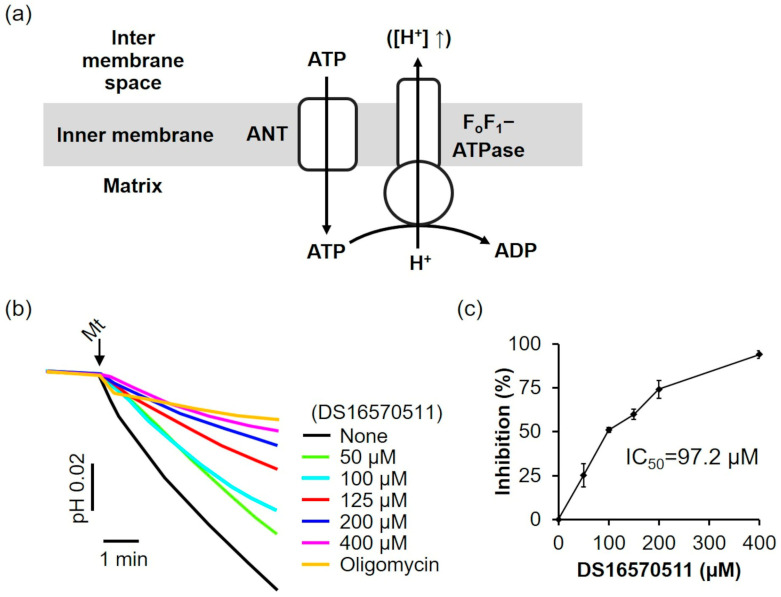
Effect of DS16570511 on mitochondrial F_o_F_1_-ATPase activity. (**a**) Schematic representation of mitochondrial F_o_F_1_-ATPase activity: ATP enters the matrix via ANT and is hydrolyzed to ADP by F_o_F_1_-ATPase, driving proton extrusion from the matrix and decreasing the reaction medium pH. The upward arrow indicates that the increase of protons in the intermembrane space is due to proton extrusion from the matrix. (**b**) Mitochondria were added to the incubation medium containing the indicated concentrations of DS16570511 or oligomycin, and pH changes in the medium were measured using a pH electrode. Representative traces are shown. (**c**) Inhibition was calculated from the pH change over a 3 min period, starting 30 s after adding mitochondria. Results are expressed as 0% inhibition in the absence of DS16570511 and 100% inhibition with oligomycin. Data are presented as the mean ± standard deviation of independent experiments (*n* = 3).

The effects of DS16570511 on F_o_F_1_-ATPase and ANT were evaluated. In the absence of a respiratory substrate and under respiratory chain inhibition, adenosine triphosphate (ATP) was transported into the mitochondria via ANT and hydrolyzed to adenosine diphosphate (ADP) by F_o_F_1_-ATP synthase. This reaction drove proton extrusion from the matrix via F_o_F_1_-ATPase, decreasing the pH of the medium (Figure 7a). The effect of DS16570511 on F_o_F_1_-ATPase/ANT was assessed by analyzing the inhibition of the pH decrease. DS16570511 inhibited the pH decrease in a concentration-dependent manner (Figure 7b), with an IC_50_ of 97.2 µM (Figure 7c). These findings suggest that DS16570511 acts nonspecifically on a broad range of proteins within the inner mitochondrial membrane.

In general, MCU facilitates Ca^2+^ uptake using the membrane potential generated by proton pumping through the respiratory chain during substrate oxidation. However, MCU can also utilize the membrane potential formed by proton pumping associated with ATP hydrolysis by F_o_F_1_-ATPase for Ca^2+^ uptake (Supplementary Figure S1a). To evaluate the effects of DS16570511 on this process, we analyzed Ca^2+^ uptake mediated by F_o_F_1_-ATPase-driven membrane potential. DS16570511 inhibited Ca^2+^ uptake in a concentration-dependent manner, similar to its effect on Ca^2+^ uptake driven by respiratory chain-dependent membrane potential (Supplementary Figure S1b). The IC_50_ of DS16570511 for Ca^2+^ uptake was 57.7 µM (Supplementary Figure S1c).

### 2.5. Structure–Activity Relationships of DS16570511

**Figure 8:**
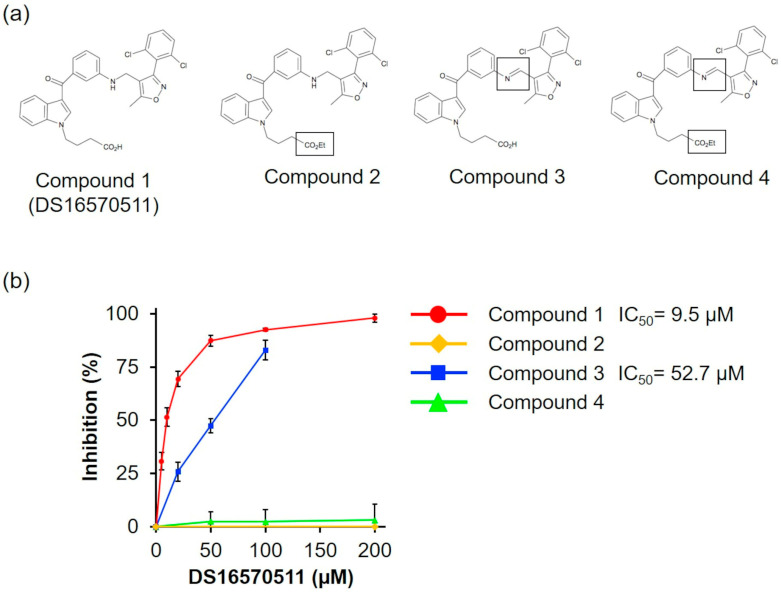
Comparison of the effects of DS16570511 analogs on oxygen consumption. (**a**) Molecular structures of DS16570511 (compound **1**) and its analogs. Compounds **2**–**4** include substitutions, as indicated by the labeled structures in the figure. (**b**) Degree of inhibition of respiratory chain complex II by each compound. The degree of inhibition was calculated as described in Figure 5. Data are presented as the mean ± standard deviation of independent experiments (*n* = 3).

To investigate the relationship between the molecular structure of DS16570511 (compound **1**) and its effect on mitochondria, we synthesized three analogs: compound **2** (with the carboxyl group replaced by an ethyl group), compound **3** (with the amino group replaced by an imino group), and compound **4** (with both substitutions) (Figure 8a). We analyzed the effects of compounds **1**–**4** on oxygen consumption via respiratory chain complex II using succinate as a respiratory substrate, as DS16570511 exhibited the most potent effect on this process among various mitochondrial functions (Figure 5, IC_50_ = 9.5 μM). We found that compound **3** exhibited reduced inhibitory activity compared with DS16570511 (IC_50_ = 52.7 µM), whereas compounds **2** and **4** completely lacked inhibitory activity (Figure 8b). These results suggest that the terminal carboxyl group plays a critical role in the inhibitory activity of DS16570511.

## 3. Discussion

The mechanism by which DS16570511 inhibits mitochondrial Ca^2+^ uptake remains controversial. Kon et al., who first reported DS16570511, demonstrated that it inhibits mitochondrial Ca^2+^ uptake without affecting mitochondrial membrane potential. Conversely, Payne et al. showed that DS16570511 affects not only Ca^2+^ uptake but also membrane potential [27]. They further reported that cyclosporin A influences the function of DS16570511. Although how cyclosporin A affects DS16570511′s activity has not been analyzed, this finding suggests that DS16570511 interacts with components of the mitochondrial permeability transition pore, such as ANT [29], cyclophilin D [7], and F_o_F_1_-ATPase [5]. Using mitochondria isolated from rat brains, Belosludtsev et al. demonstrated that at concentrations at which DS16570511 inhibits Ca^2+^ uptake, it does not suppress respiratory activity but inhibits F_o_F_1_-ATPase or ANT activity [28]. This suggests that in brain mitochondria, DS16570511 inhibits Ca^2+^ uptake not as a secondary effect of membrane potential suppression caused by respiratory inhibition but rather through direct effects on F_o_F_1_-ATPase, ANT, and other mitochondrial components, including the MCU complex. However, to the best of our knowledge, no further detailed studies on the inhibitory mechanism of DS16570511 have been published. To better understand how DS16570511 inhibits mitochondrial Ca^2+^ uptake, it is crucial to determine its inhibitory potency on individual mitochondrial functions, such as Ca^2+^ uptake, membrane potential formation, respiratory chain activity, and F_o_F_1_-ATPase/ANT activity, and to analyze their correlations. In this study, we compared the IC_50_ values of DS16570511 for various mitochondrial functions to elucidate its inhibitory mechanisms.

We examined the effects of DS16570511 on rat liver mitochondria using succinate as the respiratory substrate. DS16570511 inhibited oxygen consumption (IC_50_ = 9.5 µM) and membrane potential formation (IC_50_ = 10.8 µM). The degree of inhibition (IC_50_) for these effects was nearly identical to that for DS16570511′s effect on mitochondrial Ca^2+^ uptake (IC_50_ = 9.2 µM). These findings indicate that the inhibition of Ca^2+^ uptake by DS16570511 in succinate-energized liver mitochondria results from the inhibition of electron transfer from succinate in the respiratory chain, leading to subsequent inhibition of membrane potential formation. To investigate how DS16570511 inhibits oxygen consumption, we analyzed its effects in mitochondria supplied with electrons from complex I using glutamate and malate as respiratory substrates. DS16570511 inhibited oxygen consumption with an IC_50_ of 40.9 µM (Figure 6). This result suggests that the IC_50_ value for the most DS16570511-sensitive process in electron transfer from glutamate/malate to complexes I, III, and IV is 40.9 µM. Conversely, our findings revealed that DS16570511 exhibits a much stronger inhibitory effect on electron transfer from succinate through complexes II, III, and IV, with an IC_50_ of 9.5 µM (Figure 5). These results indicate that DS16570511 has the most potent inhibitory effect on complex II among the respiratory chain complexes.

DS16570511 inhibited F_o_F_1_-ATPase/ANT activity (Figure 7), consistent with the findings of Belosludtsev et al. In the present study, the IC_50_ of DS16570511 against membrane potential formation by F_o_F_1_-ATPase/ANT was determined to be 97.2 µM. Further analysis is needed to clarify whether this value reflects the inhibition of F_o_F_1_-ATPase, ANT, or both. Because the MCU complex can utilize the membrane potential generated by proton pumping during ATP hydrolysis via F_o_F_1_-ATPase to facilitate Ca^2+^ uptake, this F_o_F_1_-ATPase-driven membrane potential provides a tool for studying the effects of DS16570511 on Ca^2+^ uptake independently of the respiratory chain (Supplementary Figure S1a). Analysis of the inhibitory effect of DS16570511 on Ca^2+^ uptake through F_o_F_1_-ATPase-driven membrane potential revealed an IC_50_ of 57.7 µM (Supplementary Figure S1b,c), which was lower than that for F_o_F_1_-ATPase/ANT activity (97.2 µM). This indicates that the IC_50_ of 57.7 µM may reflect the inhibitory effect of DS16570511 on the MCU complex, and it might imply that DS16570511 specifically targets the MCU complex under experimental conditions in which the respiratory chain is not functional, thereby inhibiting Ca^2+^ uptake into the mitochondria. Because the membrane potential in cancer cells is often generated by F_o_F_1_-ATPase [30], DS16570511 may target the MCU complex and inhibit Ca^2+^ uptake in such contexts. Consistent with this, Kim et al. reported that DS16570511 inhibited cell proliferation in glioblastoma cells by targeting the MCU complex [31], supporting its potential application in cancer research. However, further detailed analysis is required.

Our study demonstrated that DS16570511 exerts inhibitory effects on a wide range of molecules, including the mitochondrial electron transfer system and F_o_F_1_-ATPase/ANT, with varying potency depending on the target. We hypothesize that DS16570511 interacts directly with these proteins and, due to its highly hydrophobic nature, also disrupts the phospholipid layer of the inner mitochondrial membrane. This disruption likely affects membrane fluidity and lipid orientation, thereby influencing the activity of multiple membrane-associated proteins. Structure–activity analysis of DS16570511 revealed for the first time that its carboxyl group is critical for the inhibition of respiratory chain complex II, on which DS16570511 exerted the strongest inhibitory effect. We speculate that the benzene rings and other hydrophobic functional groups in DS16570511 may form hydrophobic interactions with membrane proteins and phospholipid layers, whereas the polar amino acids of target proteins might form electrostatic interactions with the negatively charged carboxyl group at the ends of the free hydrocarbon chains. Both hydrophobic and electrostatic interactions likely play a key role in inhibiting the function of the mitochondrial inner membrane. Verification of this hypothesis will require future physicochemical analyses, such as assessing affinity for proteins and phospholipid bilayers. In this study, we specifically focused on the effect of DS16570511 on oxygen consumption using succinate as a respiratory substrate, against which DS16570511 exhibited strong inhibitory activity, and we identified the structural component responsible for this effect. This finding is expected to provide fundamental data for designing DS16570511 analogs with reduced nonspecific effects. A systematic analysis of the effects of DS16570511 analogs on various mitochondrial functions is expected to further contribute to the development of clinically applicable compounds.

**Figure 9:**
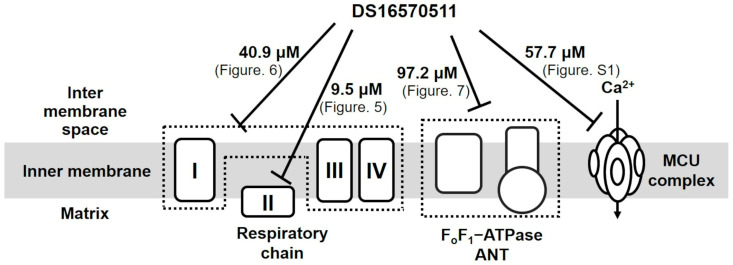
Diverse effects of DS16570511 on the function of rat liver mitochondria. DS16570511 exhibited inhibitory effects on multiple mitochondrial targets, including respiratory chain complexes, F_o_F_1_-ATPase/ANT, and the MCU complex. The IC_50_ for respiratory chain complexes I–III–IV and II was determined based on the rate of mitochondrial oxygen consumption (Figure 5 and Figure 6). The IC_50_ for F_o_F_1_-ATPase/ANT activity was calculated from mitochondrial F_o_F_1_-ATPase activity (Figure 7). The IC_50_ for the MCU complex was derived from mitochondrial Ca^2+^ uptake activity driven by ATP hydrolysis (Supplementary Figure S1).

Belosludtsev et al. investigated the effects of DS16570511 on rat brain mitochondria energized with respiratory substrates [30] and reported that its inhibitory effect on Ca^2+^ uptake was more potent than its effect on the respiratory chain. Conversely, our quantitative analysis of DS16570511′s effect on rat liver mitochondria revealed its strongest inhibitory effect on respiratory chain complex II, with an IC_50_ of 9.5 µM. This inhibitory effect was comparable to DS16570511′s effect on mitochondrial Ca^2+^ uptake (IC_50_ = 9.2 µM). DS16570511 weakly inhibited other targets, including respiratory chain complexes I–III–IV (IC_50_ = 40.9 µM), F_o_F_1_-ATPase/ANT (IC_50_ = 97.2 µM), and the MCU complex (IC_50_ = 57.7 µM) (Figure 9).

The differing intensity of DS16570511′s effects on mitochondrial functions between liver and brain mitochondria remains unclear. Because DS16570511 appears to broadly target mitochondrial membrane proteins and phospholipid layers, its effects may be influenced by the proteome and lipid composition of mitochondrial membranes. Notably, liver and brain mitochondria have distinct proteomic profiles [32] and differences in the lipid composition of their phospholipid layers, which could explain the tissue-specific variation in DS16570511′s effects. Further analysis is needed to elucidate these differences.

## 4. Materials and Methods

### 4.1. Synthesis of DS16570511 and Its Analogs

DS16570511 and its analogs were synthesized as described by Kon et al. Detailed synthesis procedures are provided in the Supplementary Information [33,34].

### 4.2. Isolation of Rat Liver Mitochondria

Livers were harvested from 6- to 18-week-old male Wistar rats (Japan SLC, Shizuoka, Japan), and mitochondria were isolated as previously described [35]. All animal experiments complied with the University of Tokushima Guidelines for the Care and Use of Laboratory Animals (approval number: T28-29). Mitochondrial protein concentrations were measured using the Biuret method with bovine serum albumin as the standard in the presence of 1% SDS.

### 4.3. Mitochondrial Ca^2+^ Uptake Assay

Fluo-5 (1 µM) was used as a Ca^2+^-sensitive fluorescence indicator in 1 mL of Pi medium (250 mM sucrose, 10 mM Tris-MOPS, 10 µM EGTA-Tris, 0.5 mg/mL bovine serum albumin, and 10 mM KPi, pH 7.4). Fluorescence intensity was measured using an F-2700 spectrofluorometer (HITACHI, Tokyo, Japan) with excitation and emission wavelengths of 494 and 516, respectively. The Pi medium was premixed with 2.5 µM CsA, 0.68 µg/mL rotenone, 10 mM succinate, 100 µM CaCl_2_, and DS16570511. Mitochondria were suspended at 0.42 mg protein/mL for measurements. To assess F_o_F_1_-ATPase-driven Ca^2+^ uptake activity, Calcium Green-5N (1 µM) was used as a Ca^2+^-sensitive fluorescence indicator (Ex/Em = 506/532 nm), and 1 mM ATP was added as a substrate to generate the membrane potential. Measurements were conducted in the presence of 2.5 µM antimycin and 0.68 µg/mL rotenone as respiratory chain inhibitors. The degree of Ca^2+^ uptake inhibition by DS16570511 and IC_50_ values were calculated based on the rate of Ca^2+^ uptake 30 s after adding mitochondria.

### 4.4. Measurement of Mitochondrial Membrane Potential

DiSC_3_(5) (3 µM) was used as a fluorescent probe to measure mitochondrial membrane potential in 2.2 mL of Pi medium. Fluorescence intensity was measured using an F-2700 spectrofluorometer (HITACHI, Tokyo, Japan) with excitation and emission wavelengths of 643 and 666 nm, respectively. Fluorescence intensity was recorded after adding 10 mM succinate to Pi medium containing 2.5 µM CsA, 0.68 µg/mL rotenone, 0.42 mg protein/mL mitochondria, and DS16570511. The degree of inhibition of membrane potential formation by DS16570511 and IC_50_ values were determined based on the rate of membrane potential formation 5 s after succinate addition.

### 4.5. Measurement of the Rate of Mitochondrial Oxygen Consumption

The rate of mitochondrial oxygen consumption was measured using a Clark-type oxygen electrode (YSI5331, Yellow Springs Instrument Co., Ltd., Yellow Springs, OH, USA) at 25 °C in 2.2 mL of Pi medium preincubated with 2.5 µM CsA. When electrons were supplied via respiratory chain complex I, 5 mM glutamate and 5 mM malate were used as respiratory substrates. For electrons supplied via respiratory chain complex II, 0.68 µg/mL rotenone was added beforehand to inhibit electron transfer from endogenous respiratory chain complex I, and 10 mM succinate was used as the respiratory substrate. The degree of inhibition and IC_50_ values of DS16570511 were calculated based on the oxygen consumption rate over a 3 min period, starting 30 s after the addition of SF6847.

### 4.6. Measurement of Mitochondrial F_o_F_1_-ATPase Activity

Mitochondrial F_o_F_1_-ATPase activity was measured using a modified version of the classical pH electrode-based method [36]. The assay was conducted in 2.2 mL of incubation medium containing 200 mM sucrose, 20 mM KCl, 3 mM MgCl_2_, and 3 mM KPi (pH 7.4), preincubated with 2.5 µM CsA, 2.5 µM antimycin, 1 mM ATP, and DS16570511. The pH was adjusted to 7.4 using potassium hydroxide or HCl. Mitochondria were suspended at a protein concentration of 0.42 mg/mL, and the assay was performed at 25 °C. The degree of inhibition of FoF1-ATPase activity by DS16570511 and IC_50_ values were determined based on the pH change over a 3 min period, starting 30 s after adding mitochondria.

## 5. Conclusions

In this study, we conducted a quantitative biochemical analysis of the effects of DS16570511 on mitochondrial functions, including Ca^2+^ uptake, using rat liver mitochondria. We calculated the IC_50_ values of DS16570511 for each function and compared them. The following results were obtained: (i) DS16570511 inhibits a wide range of proteins with varying degrees of potency; (ii) among its broad range of targets, DS16570511 particularly inhibits respiratory chain complex II, and this inhibition indirectly suppresses mitochondrial Ca^2+^ uptake by preventing membrane potential formation; and (iii) the carboxyl group at the terminus of DS16570511 is critical for its ability to inhibit mitochondrial Ca^2+^ uptake through respiratory chain complex II inhibition. These findings enhance our understanding of the mechanism by which DS16570511 inhibits mitochondrial Ca^2+^ uptake and provide valuable insights for developing drugs targeting mitochondrial Ca^2+^ regulation while considering potential off-target effects. Because mitochondrial Ca^2+^ uptake is implicated in various pathological conditions and diseases, including ischemia–reperfusion injury, muscular dystrophy, neurodegenerative diseases, and cancer, identifying effective inhibitors is crucial for advancing clinical applications.

## Data Availability

All data are contained within the article or its Supplementary Information.

## Supplementary Materials

The following supporting information can be downloaded at: https://www.mdpi.com/article/10.3390/ijms26062670/s1.

## Abbreviations

The following abbreviations are used in this manuscript:

ANT	Adenine nucleotide translocator
ATP	Adenosine triphosphate
ADP	Adenosine diphosphate
EMRE	Essential MCU regulator
MCU	Mitochondrial calcium uniporter
MICU1	Mitochondrial calcium uptake 1
