# Supplementary material for: Multiple Inhibitory Mechanisms of DS16570511 Targeting Mitochondrial Calcium Uptake: Insights from Biochemical Analysis of Rat Liver Mitochondria
Source: Int J Mol Sci. 2025 Mar 16;26(6):2670. doi: 10.3390/ijms26062670 (PMC11942279; doi:10.3390/ijms26062670)
Supplement: Supplementary file 1 [file ijms-26-02670-s001.zip › Supplemental Figure.pdf]

Supplemental Figure 1

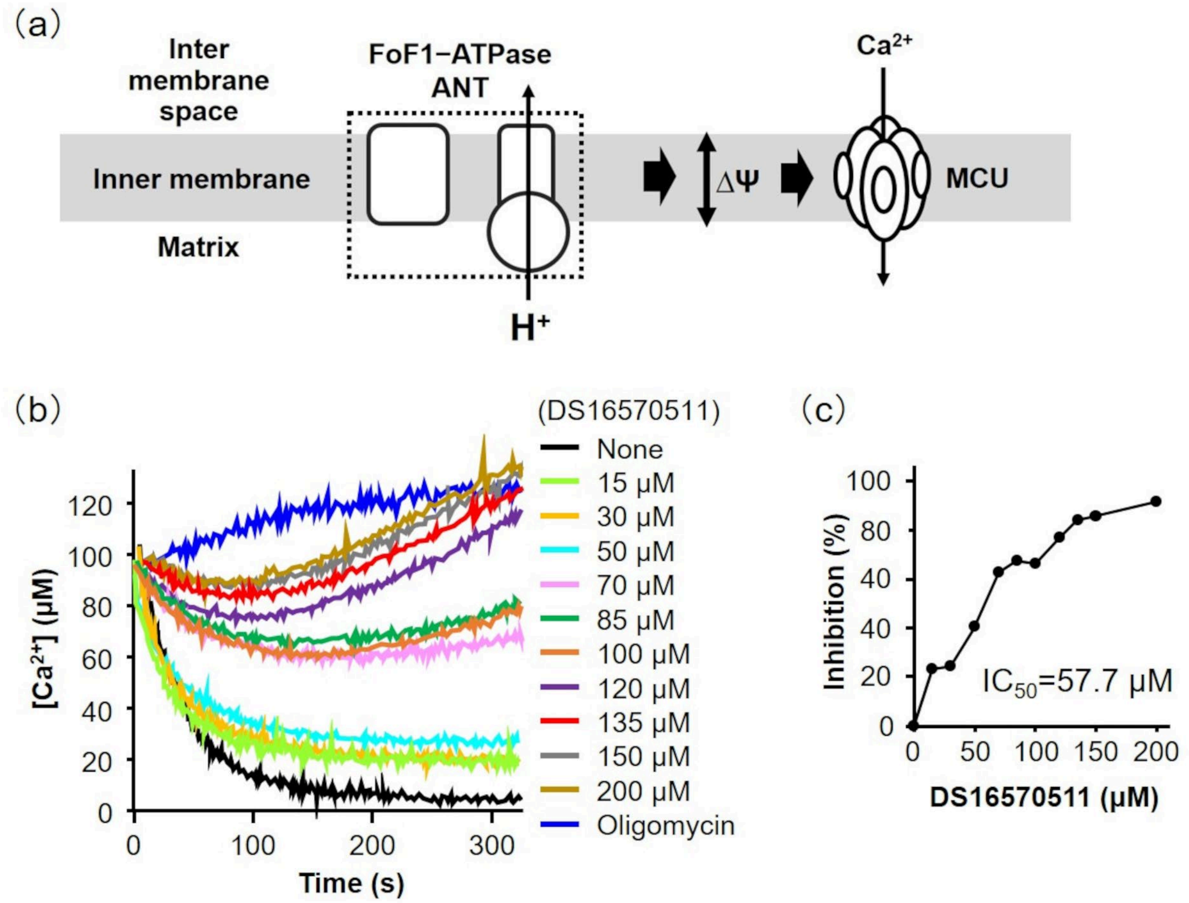

Supplemental Figure 1. Effect of DS16570511 on mitochondrial Ca^{2+} uptake driven by F_oF_1-ATPase-generated membrane potential. (**a**) Schematic of ATP-driven mitochondrial Ca^{2+} uptake: F_oF_1-ATPase hydrolyzes ATP transported into the matrix via ANT, generating membrane potential through proton extrusion, which facilitates Ca^{2+} uptake via MCU. (**b**) Isolated mitochondria were added to the Pi medium containing the indicated concentrations of DS16570511 or oligomycin. Changes in extra-mitochondrial Ca^{2+} concentration over time were measured using Calcium Green-5N fluorescence intensity. (**c**) Inhibition was quantified based on the Ca^{2+} uptake rate during the first 30 s after mitochondria addition, with 0% representing the control (no DS16570511) and 100% representing oligomycin.
