# Supplementary material for: Multiple Inhibitory Mechanisms of DS16570511 Targeting Mitochondrial Calcium Uptake: Insights from Biochemical Analysis of Rat Liver Mitochondria
Source: Int J Mol Sci. 2025 Mar 16;26(6):2670. doi: 10.3390/ijms26062670 (PMC11942279; doi:10.3390/ijms26062670)
Supplement: Supplementary file 1 [file ijms-26-02670-s001.zip › Supplemental Information.pdf]

1. General procedure

All reactions were conducted under a positive argon pressure. Analytical thin-layer chromatography was performed on Merck TLC silica gel 60F254 plates. Visualization was achieved with molibudenium phosphate, *p*-anisaldehyde, Hannessian's cocktail, or ninhydrin. For column chromatography, silica gel (KANTO KAGAKU N-60) was employed. NMR spectra were recorded using a Bruker AV400N at 400 MHz for ^{1}H and a JEOL JNM-AL300 at 75 MHz for ^{13}C, using the specified solvents as well as tetramethylsilane as the internal standard. Chemical shifts were reported in parts per million on the δ scale relative to the internal standard (NMR descriptions: s, singlet; d, doublet; t, triplet; q, quartet; qn, quintet; m, multiplet). Coupling constants, *J*, are reported in Hertz. Mass spectra were recorded on a Waters MICROMASS® LCT PREMIERTM (ESI-TOF), and IR spectra were measured on a JEOL FT-IR 6200. For product analysis, a Cosmosil 5C_{18}-AR-II analytical column (4.6 × 250 mm; flow rate, 1.0 mL/min; Nacalai Tesque) was employed, with the eluting product detected via UV at 220 nm. For HPLC elution, the solvent system comprised 0.1% TFA aqueous solution (v/v, solvent A) and 0.1% TFA in MeCN (v/v, solvent B). Materials were purchased from Tokyo Chemical Industry Co., Ltd., Sigma-Aldrich Inc., Wako Pure Chemical Industries Ltd., Nacalai Tesque Inc., and Kanto Chemical Co., Inc., and they were used without further purification.

2. Synthesis of the compounds

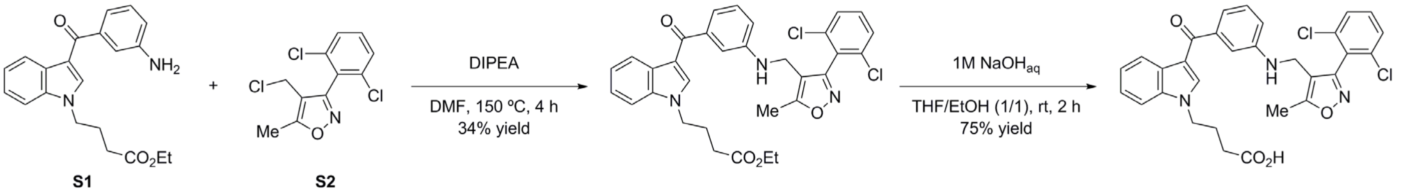

Ethyl 4-(3-(3-(((3-(2,6-dichlorophenyl)-5-methylisoxazol-4-yl)methyl)amino)benzoyl)-1*H*-indol-1-yl)butanoate

A solution of **S1** [41] (287 mg, 0.818 mmol), **S2** [42] (269 mg, 0.971 mmol), and DIPEA (0.214 mL, 1.23 mmol) in DMF (4.0 mL) was stirred at 150 ºC for 4 h. Subsequently, the mixture was evaporated *in vacuo* and purified via silica gel column chromatography (hexane/EtOAc = 3/1 to 3/2), resulting in the desired product (235 mg, 34%): a yellow amorphous solid. Retention time = 12.0 min (analytical HPLC conditions: linear gradient of solvent B in solvent A, 65%–95% over 30 min); ^{1}H NMR (400 MHz, $CDCl_3$) δ 8.41 (m, 1H), 7.52 (s, 1H), 7.43–7.27 (m, 6H), 7.20 (dd, *J* = 7.6, 7.6 Hz, 1H), 7.11 (d, *J* = 7.6 Hz, 1H), 6.95 (m, 1H), 6.66 (dd, *J* = 8.0, 1.6 Hz, 1H), 4.23 (t, *J* = 6.8 Hz, 2H), 4.08 (q, *J* = 7.2 Hz, 2H), 4.01 (s, 2H), 3.81 (s, 1H), 2.52 (s, 3H), 2.30 (t, *J* = 6.8 Hz, 2H), 2.18 (qn, *J* = 6.8 Hz, 2H), 1.20 (t, *J* = 7.2 Hz, 3H); ^{13}C NMR (75.0 MHz, $CDCl_3$) δ 191.0, 172.4, 167.7, 159.2, 147.5, 141.8, 136.8, 136.7, 135.5, 131.3, 128.9, 128.1, 127.9, 127.3, 123.6, 122.8, 122.6, 118.5, 115.8, 115.7, 112.3, 109.8, 60.7, 46.0, 36.9, 30.8, 25.0, 14.1, 11.5; IR (KBr) ν 3362, 2980, 1727, 1626, 1198, 1166 cm^{-1}; HRMS-ESI (*m/z*): [M + H$^+$] calculated for $C_{32}H_{30}$ $Cl_2N_3O_4$; 590.1613, found, 590.1594.

4-(3-(3-(((3-(2,6-Dichlorophenyl)-5-methylisoxazol-4-yl)methyl)amino)benzoyl)-1*H*-indol-1-yl)butanoic acid

To s solution of **2** (179 mg, 0.301 mmol) in THF (1.5 mL) and EtOH (1.5 mL) was added 1 M NaOHaq (1.50 mL, 1.50 mmol) at room temperature and stirred for 2 h. Subsequently, the reaction was quenched with 5% $KHSO_4$(aq) at 0 ºC, extracted with EtOAc, dried over Na_2SO_4, and evaporated *in vacuo*, yielding the desired product (128 mg, 75%): a yellow amorphous solid. Retention time = 27.8 min (analytical HPLC conditions: linear gradient of solvent B in solvent A, 5%–95% over 30 min); ^{1}H NMR (400 MHz, DMSO-d_6) δ 12.2 (s, 1H), 8.25 (d, *J* = 7.8 Hz, 1H), 7.84 (s, 1H), 7.65 (d, *J* = 7.8 Hz, 1H), 7.54–7.41 (m, 3H), 7.33 (dd, *J* = 7.3, 7.3 Hz, 1H), 7.27 (dd, *J* = 7.8, 7.8 Hz, 1H), 7.16 (dd, *J* 7.8, 7.8 Hz, 1H), 6.93 (d, *J* = 7.4 Hz, 1H), 6.77 (s, 1H), 6.65 (d, *J* = 7.8 Hz, 1H), 6.04 (t, *J* = 5.6 Hz, 1H), 4.28 (t, *J* = 6.6 Hz, 2H), 3.98 (d, *J* = 5.6 Hz, 2H), 2.23 (t, *J* = 6.6 Hz, 2H), 2.00 (qn, *J* = 6.6 Hz, 2H) (the signal of oxazole's methyl group was presumed to overlap with the residual solvent

peak); ^{13}C NMR (75.0 MHz, $CDCl_3$) δ 191.2, 177.4, 159.3, 147.3, 141.6, 136.9, 136.7, 135.5, 131.4, 128.9, 128.1, 127.8, 127.2, 123.7, 122.9, 122.7, 118.6, 115.8, 112.4, 109.7, 45.9, 36.9, 30.5, 24.7, 11.4; IR (KBr) ν 3406, 3384, 2982, 1726, 1623, 1198, 1168 cm^{-1}; HRMS-ESI (*m/z*): $[M + K^+]$ calculated for $C_{30}H_{25}Cl_2N_3O_4$; 600.0859, found, 600.0839.

3. ^1H- and ^{13}C-NMR spectra

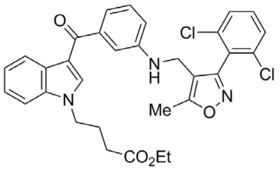

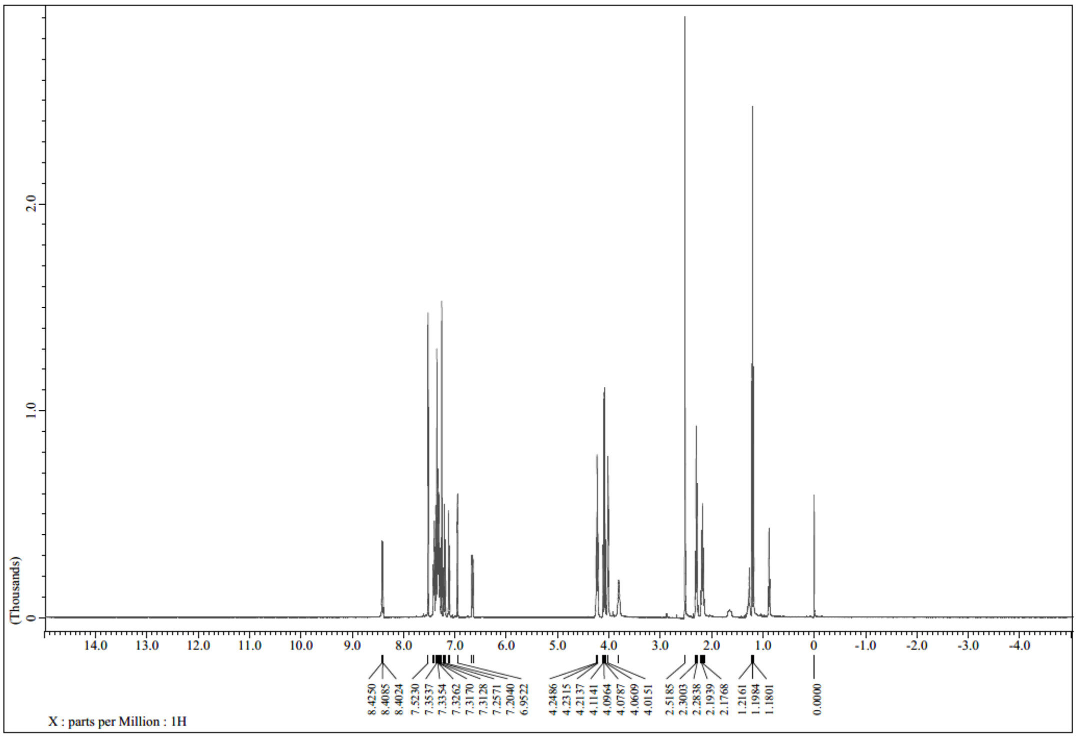

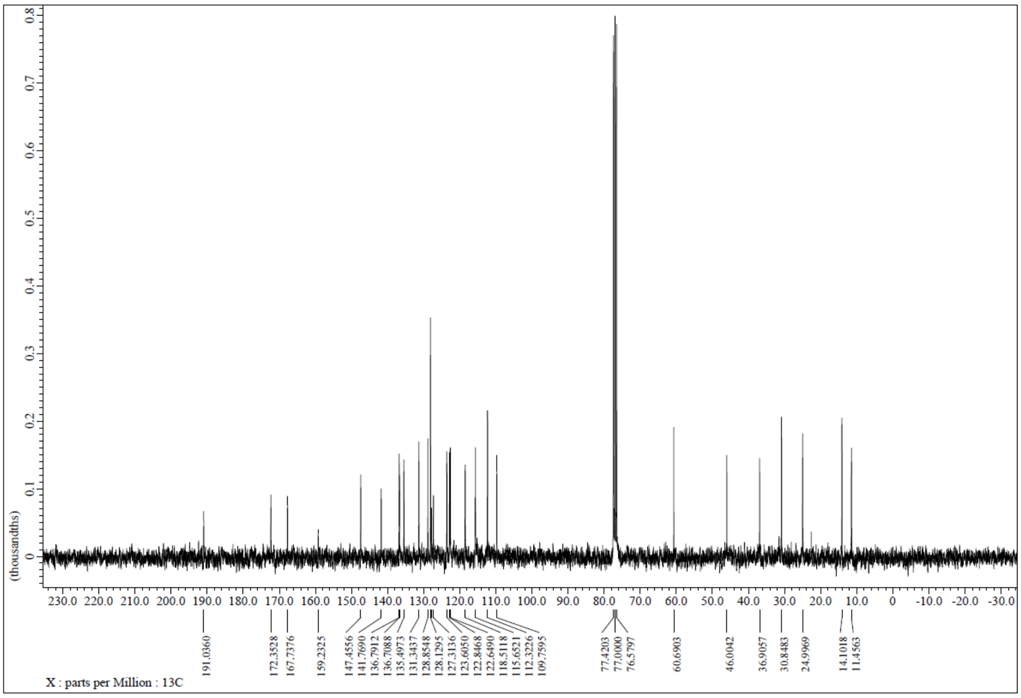

X : parts per Million : 13C
(thousandths)

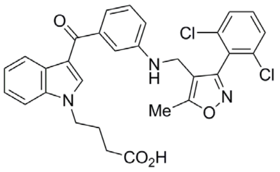

Cl
Cl
N
H
Me
O
N
CO2H

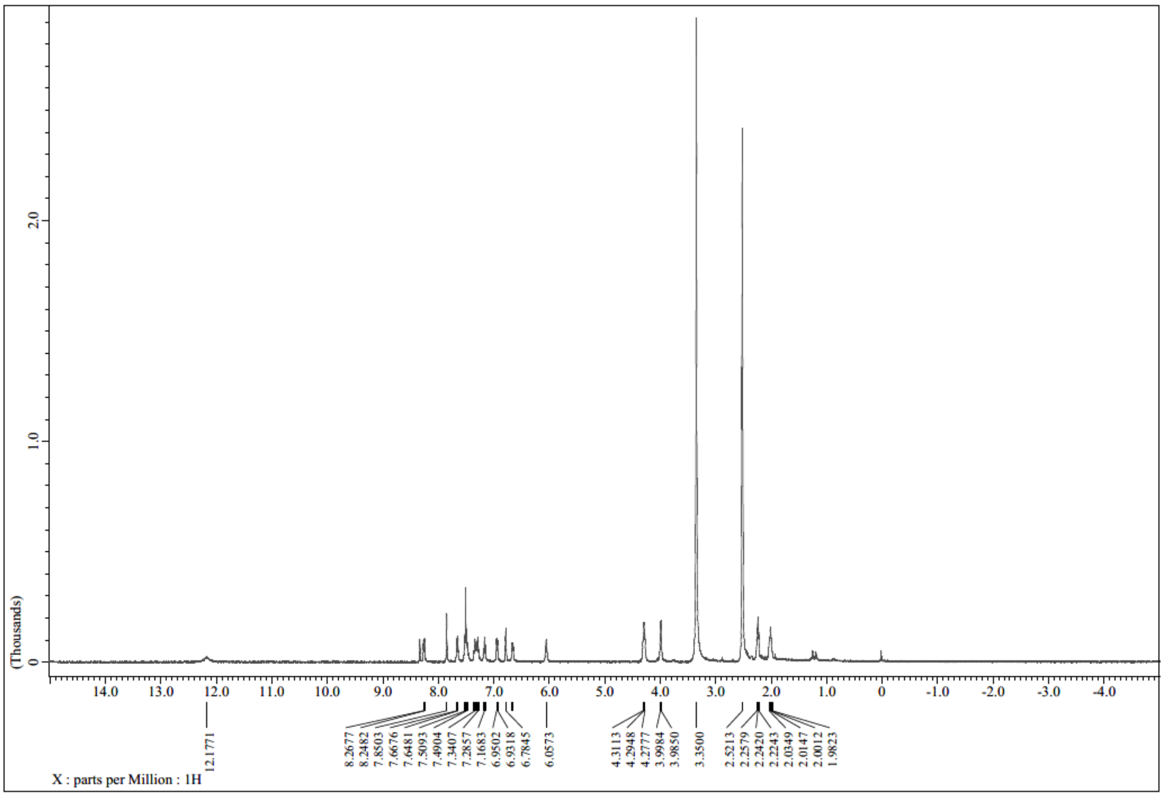

X : parts per Million : 1H
(Thousands)

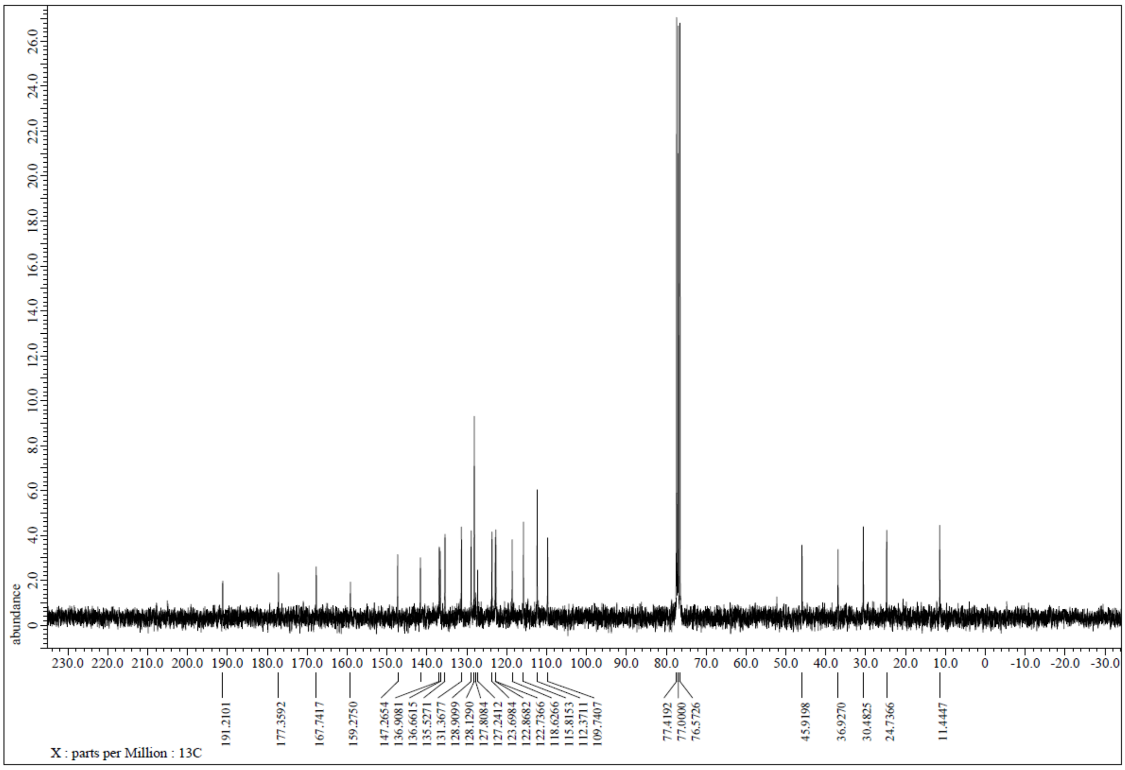

4. HPLC chart

Analytical HPLC conditions: linear gradient of solvent B in solvent A, 65%–95% over 30 min.

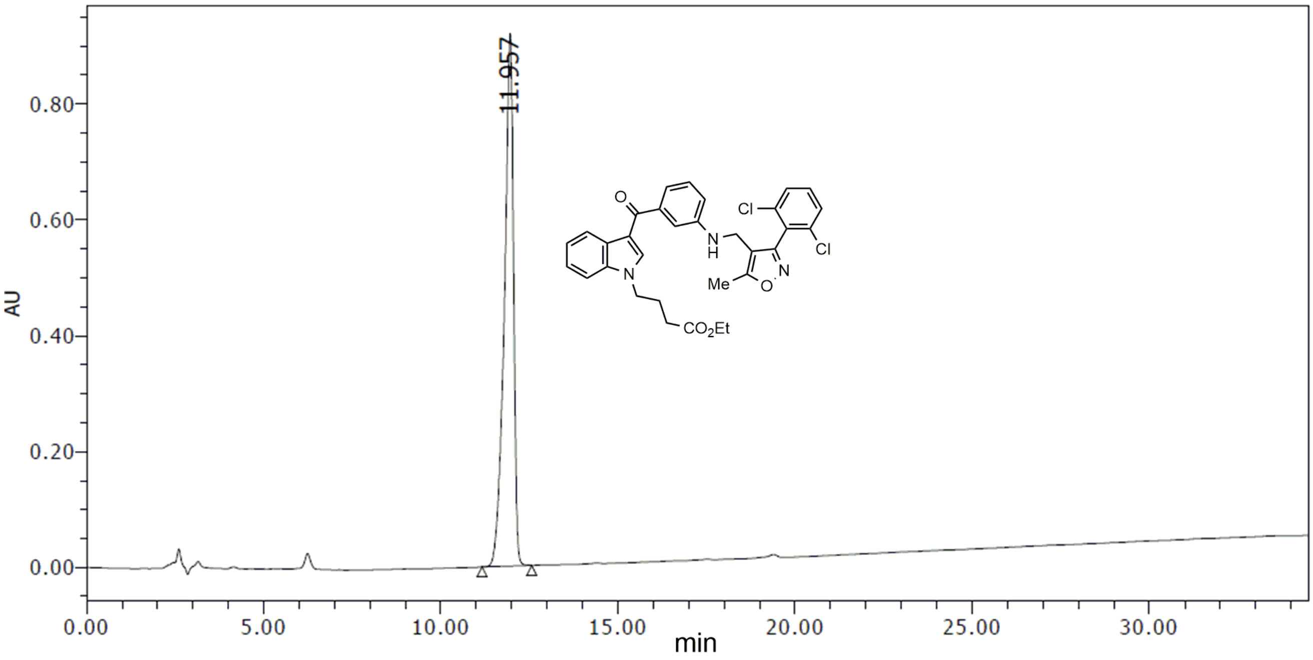

Analytical HPLC conditions: linear gradient of solvent B in solvent A, 5%–95% over 30 min.

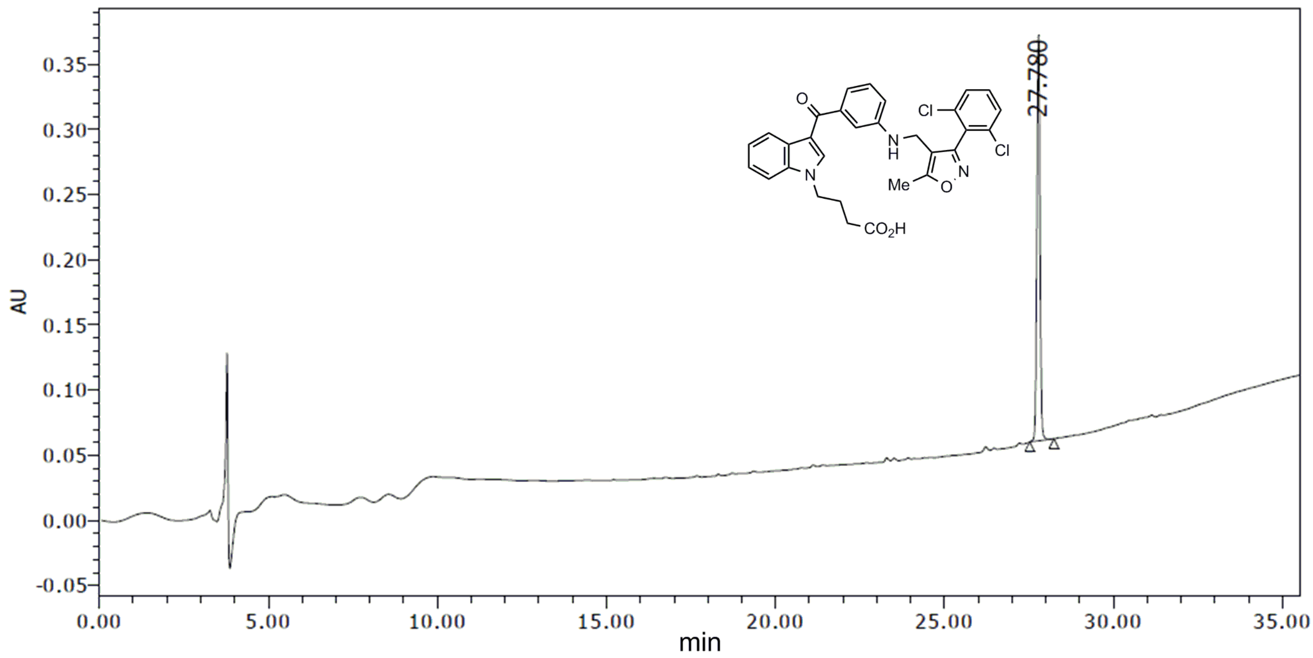

27.780
O
N
H
Cl
Cl
N
Me
O
N
CO2H
AU
0.35
0.30
0.25
0.20
0.15
0.10
0.05
0.00
-0.05
0.00
5.00
10.00
15.00
20.00
25.00
30.00
35.00
min
